# Whole-Genome Sequence of an Epidemic Strain of *Burkholderia pseudomallei* vgh07 in Taiwan

**DOI:** 10.1128/genomeA.00345-15

**Published:** 2015-04-30

**Authors:** Yao-Shen Chen, Hsi-Hsun Lin, Pei-Tan Hsueh, Pei-Ju Liu, Wen-Fan Ni, Wan-Chia Chung, Chih-Peng Lin, Ya-Lei Chen

**Affiliations:** a Division of Infectious Diseases, Kaohsiung Veterans General Hospital, Kaohsiung, Taiwan; b Department of Internal Medicine, National Yang-Ming University, Taipei, Taiwan; c Department of Medicine, Section of Infectious Disease, E-Da Hospital, Kaohsiung, Taiwan; d Department of Biological Science, National Sun Yat-sen University, Kaohsiung, Taiwan; e Department of Biotechnology, National Kaohsiung Normal University, Kaohsiung, Taiwan; f Bioinformatics Division, Yourgene Bioscience Inc., New Taipei City, Taiwan

## Abstract

Here, we report the complete genome sequence of *B. pseudomallei* vgh07. This is an epidemic strain that was isolated from a melioidosis patient with arthro-osteomyelitis in Taiwan.

## GENOME ANNOUNCEMENT

Melioidosis is a fatal infectious disease that is caused by the saprophyte *Burkholderia pseudomallei* in areas in which it is endemic, such as northern Australia, Thailand, and Taiwan, but its occurrence is most likely underestimated elsewhere (1). Clinically, the symptoms of melioidosis vary; septic melioidosis, concomitant pneumonia, and splenic and hepatic abscess are commonly seen (2). However, due to geographical seclusion, the genomes of *B. pseudomallei* isolates evolved independently, resulting in different manifestations of melioidosis in separated regions. For example, there were more cases with hepatosplenic suppuration in Thailand, whereas more cases displayed prostatic abscesses and encephalomyelitis in Australia (3). Deciphering whole-genomic sequences provides insight into the pathogen’s phylogeny or diversity and the biogeographical contribution of virulence; however, the majority of the currently available sequences are from *B. pseudomallei* strains isolated in Thailand or northern Australia (4, 5).

*B. pseudomallei* vgh07 is one of the epidemic strains (ST58, by multilocus sequence typing) in Taiwan (6). It was isolated from the blood of a melioidosis patient with septic arthro-osteomyelitis (7). The genomes were extracted by mini-QIAamp DNA isolation kits (Qiagen, Germany) and then sequenced using a combination of HiSeq 2500 System (Illumina, Inc., San Diego, CA, USA) and PacBio (Pacific Bioscience of California, Inc., Menlo Park, CA, USA) technologies. On average, 131 bp of the paired-end (insert size, 183 bp) and 136 bp of the mate-pair (insert size, 4,300 bp) Illumina sequencing libraries and a PacBio sequencing library (insert size, >20 kb) were generated. From the Illumina sequencing reads, 12 and 117 scaffolds were assembled using the ALLPATH-LG version 47655 and Velvet version 1.2.09 programs, respectively. For PacBio technology, the consensus sequence contigs were assembled from long reads using the HGAP assembler. A physical map of the *B. pseudomallei* vgh07 genome was drafted using the Argus optical mapping system (OpGen, Inc., Gaithersburg, MD, USA) with BamHI digestion. Using MapSolver software (OpGen), all of the scaffolds and contigs were matched and connected. The locations were then mapped using the Minimus2 program. In total, 51 of the gaps were filled using GapCloser version 1.12. Twelve of the unclosed gaps with a total length of 102,314 bp were filled by using the HGAP assembly results or by using PCR and Sanger sequencing methods.

*B. pseudomallei* vgh07 consists of two chromosomes. Chromosome 1 is 4,006,427 bp in size (G+C content, 67.9%), with 3,384 protein-coding sequences, 9 rRNA clusters, and 52 tRNA genes; chromosome 2 is 3,039,355 bp in size (G+C content, 68.6%), with 2,312 protein-coding sequences, 3 rRNA clusters, and 7 tRNA genes. A total of 52 hypothetical proteins were unique to the NCBI protein database (e-value cutoff, 10^−5^). By insertion sequence (IS) finder analysis (https://www-is.biotoul.fr), IS407 elements were inserted into chromosomes 1 (*n* = 3) and 2 (*n* = 3). Moreover, ISBam1, ISBam2, ISBam3, and ISRso15 were estimated on chromosomes 1 and 2, whereas ISRso8 and ISBps1 were found only on chromosome 2.

### Nucleotide sequence accession numbers.

The whole-genome sequences have been deposited at GenBank under the accession numbers CP010973 (chromosome 1) and CP010974 (chromosome 2).
